# Modified Inoculation Preferable to Vaccination

**Published:** 1899-05

**Authors:** C. H. Tebault

**Affiliations:** Surgeon General of the United Confederate Veterans


### Abstracts and Selections.

### Modified Inoculation Preferable to Vaccination.

BY C. H. TEBAULT, M. D.,
Surgeon General of the United Confederate Veterans.

At the Nashville meeting of the United Confederate Veterans, Surgeon General Tebault submitted a report on modified inoculation which deserves wider publicity among the medical profession than it received by publication in the report of the proceedings. For that reason we print the report below in full:

SURGEON GENERAL'S REPORT.

*Headquarters United Confederate Veterans,*
*Surgeon General's Office.*

NEW ORLEANS, LA., June 17, 1897.

*Major General Geo. Moorman, Adjutant General and Chief of Staff U. C. V.'s, New Orleans, La.*

GENERAL:—I have the honor to submit that as outlined in my report at the Sixth Annual Reunion, I expected on this occasion to offer a very full statement connected with my department. The great Lafayette Square fire of the 15th of April just passed, involved my own residence, and so scattered and destroyed the data I had collected for that purpose, that I have been compelled to turn from that determination, and to report upon another subject, which, though largely personal, possesses, in my judgment, a wide and general interest.

In pursuance of this object, I beg to invite attention to the following, which I borrow from:

"The military operations of General Beauregard, in the war between the States, 1861 to 1865, etc., by Alfred Roman." in Volume I, pages pages 372 and 373:

"On the 20th and 22nd of May, General Villepigue informed General Beauregard that the enemy had sent to Fort Pillow two hundred prisoners, most of whom were sick with smallpox, and who had been received without his authority, by the second officer in command. Believing, as did also General Villepigue, that this would result in communicating that terrible disease to the garrison, and thereby destroy its effectiveness, General Beauregard at once telegraphed, 'return them forthwith.' But Commodore Davis, of the United States Navy, peremptorily refused to take them back. They were cared for by General Villepigue, and placed with great difficulty, in separate quarters, under the intelligent and devoted supervision of Doctor C. H. Tebault, of Louisiana, then a surgeon in the Confederate Army. He wrote an interesting paper on the subject, detailing all the circumstances; but this document, to our regret, is not in our possession."

My distinct recollection of the facts connected with the above quotation is, that General Beauregard had sent to General Halleck, via Corinth, two hundred and two Federal prisoners, and that by way of Fort Pillow, through Commodore Davis, the same number of Confederate prisoners had been returned in exchange by General Halleck. On reaching Fort Pillow, under the flag of truce, these exchanged Confederate prisoners were reported to be at that moment suffering from smallpox. When Brigadier General J. B. Villepigue, who had been temporarily absent, returned to his headquarters and was informed of this report regarding the state of health of these exchanged Confederates, the writer of this present report was sent for in his then capacity of Acting Medical Director of the Fort, and directed to visit these prisoners thus exchanged and report to General Villepigue their actual condition, and General Villepigue remarked, to the writer, that if they were found to be suffering from the loathsome and most contagious disease in question, he proposed to immediately return them to General Halleck. The author of this report accordingly made the visit to, and thoroughly examined these exchanged Confederate prisoners, and reported at once that General Villepigue's information with respect to the malady referred to was absolutely correct.

These exchanged Confederate prisoners stated to the writer that they had been captured at Pea Ridge, Arkansas, where Confederate Generals McIntosh and McCulloch were killed; that they numbered, when taken prisoners, something over eight hundred; that they all died at Alton, Illinois, but this remnant, from smallpox; that they had come direct from Alton, to be thus exchanged, and they concluded by imploring me to intercede in their behalf with General Villepigue, that they be not returned to what they believed would prove certain death to them, for they had learned in some manner General Villepigue's intention in the premises. The anthor did effectually intercede with General Villepigue, and the writer, accompanied by Commodore Montgomery, of the Cotton Boatram

Fleet, and General Jeff Thompson, selected Hatchie Island, between Fort Pillow and Fort Randolph, where they were placed for proper attention and treatment, and the writer volunteered to assume charge of them, and was accordingly appointed by General Villepigue in charge of them. The exchanged Federal prisoners first above alluded to were sound in body and limb, and the Confederates exchanged for these, were in all stages of that fell disease, smallpox, when received at Fort Pillow, and placed under the writer's care, with the exception of about six or seven not yet attacked. I make this above statement as a matter of history without comment. I proceed now to the kernel and objective point of my present report. Having no vaccine matter at my disposal and none within possible reach, the writer's best thoughts were taxed for some means to protect those not yet attacked, and to safeguard the garrison as well as himself. Being familiar with the great Jenner's writings in this relation, the writer recalled the fact that the true Jennerian virus was that derived from the cow while yielding milk, and after the cow had been inoculated with the grease taken from the horse, and the writer had in his then limited observation, noticed, without a failure, that it as seemingly impossible to successfully vaccinate a child exclusively fed upon good, pure cow's milk alone.

MILK AS A DILUENT.

It happened that there was a cow on this island furnishing milk, and the writer conceived the idea of admixing the smallpox lymph of the attacked prisoners with the warm milk of the cow in question, and with the thus modified smallpox lymph, to protect those not yet suffering from the malady, and protect himself as well.

The experiment proved a valuable one, for the dreaded malady was instantly arrested. The few who had escaped the smallpox responded promptly to the modified inoculation, running the same regular course observed in vaccination, and presenting all the phases of that well known operation. When Fort Pillow was evacuated, these exchanged Confederates were transported under my charge on the Paul Jones, of Commodore Montgomery's fleet to Memphis, and were finally delivered by me to General Price at Tupelo, Mississippi. The smallpox did not extend to the garrison at Fort Pillow, and was effectually arrested with these exchanged prisoners, through the protective power of this modified smallpox virus.

Some months preceding the termination of the war, and while on duty at the Hospital Post of Macon, Georgia, and assigned to the Ocmulgee Hospital, Surgeon Sanford E. Chaillé in charge, another opportunity for testing the value of modified inoculation presented. At this post, in association with my hospital duties, it became my duty to protect against the contagion of smallpox every soldier returning from this post to his command in the field, if not already sufficiently protected. The vaccine virus had become so dangerous that mothers refused to have their infants vaccinated. By this refusal, the means for propagating good vaccine virus failed to meet the demands, and here again modified inoculation was successfully evoked. At Vineville, on the edge of Macon, was located a smallpox encampment, and modified inoculation was practiced at this locality on the children and adults desiring protection, and from this encampment was procured the smallpox virus for the modified inoculation performed on the unprotected soldiers returning to the field. At this post, a soldier was not considered protected against smallpox who had not undergone a re-vaccination after the lapse of two years. Fearing the bad vaccine virus, which caused many amputations, as well as deaths, by reason of its impurity, these returning soldiers yielded without hesitation to the fresh and pure modified inoculation, which operated a complete success in every way and from every standpoint. In a hurried report of this character, the author cannot do more than thus briefly state facts, as a detailed account would make the report too lengthy for the purpose in hand.

### JENNER'S CAUTION AGAINST SPONTANEOUSLY ACQUIRED VACCINE VIRUS.

Let me briefly refer to Jenner, again, to say that he, in his day, cautioned against the employment of the vaccine virus spontaneously acquired by the cow. He designated virus thus obtained, spurious vaccine virus. The Jennerian virus was thus obtained: In England, the farriers, as well as milkmaids, indifferently milked the cows of the dairy farm. Milch cows walking or running through the fields would scratch their udders with briars thus encountered, and the farriers proceeding from the care of horses suffering with the grease, would engage in milking the cows without first washing their hands, and so communicating the matter of the grease to the scratched udder, would result in inoculating the cows, producing, in consequence, the cow pox, thus furnishing, in Jenner's view, the only reliable vaccine virus. The only kind he recommended or depended upon for protecting his patients.

Jenner observed that the milch cows suffering from the cow pox, thus acquired, furnished a reduced supply of milk, and he foresaw that when the owners of dairies understood how this cow pox was produced, that steps would be taken to avoid this inoculation from the grease of the horse to the cow, and so naturally avoid a lessening of dairy profits by reason of this disease, thus propagated. And in order to safeguard their profits, it would only be necessary to shield the cow from the grease of the horse by prohibiting farriers from milking cows, and assigning this duty only to women, as obtained in Ireland, where no cow pox prevailed in consequence of this fact.

When dairy owners should thus protect their profits, Jenner foresaw that the genuine, and, in his view, the only reliable vaccine virus would cease to exist, and that some other source would have to be provided.

The virus now employed is no longer the true Jennerian virus, but the spurious or weak virus, condemned in his day and practice. The spontaneously acquired disease is the present source from which the vaccine virus now used is obtained—the source specially condemned by Jenner, as too weak to be depended upon for continued protection against smallpox. Not to extend this report beyond a reasonably readable length, I will conclude at this point by summarizing the advantages offered by modified inoculation:

### ADVANTAGES OF MODIFIED INOCULATION.

1. Simultaneously with the presence of smallpox, we have offered us the means for arresting the disease in its first appearance by effectually limiting it to the first cases presenting.

2. No doubt could exist with respect to its strength or freshness, for the physician can thus escape the intermediary, and estimate, in his own knowledge, its freshness in exact minutes and hours.

3. Should a father enter his own home attacked by smallpox, every member of his family could be protected through him, and no questioning would be necessary, in employing the virus for modified inoculation taken from himself, for the protection of his own family.

4. Modified inoculation protects more rapidly than the best possible vaccine virus and more certainly, for the author, and every practitioner of medicine of ripe experience and who has seen much of smallpox, knows that smallpox has repeatedly overtaken vaccination two weeks after its successful insertion, and even later, while in the author's experience, modified inoculation has arrested smallpox already in the papular stage.

5. Modified inoculation would make it unnecessary to provide for compulsory vaccination, when no physician employing the humanized, or the bovine virus, can vouch, personally, for its freshness or its purity.

6. Today every physician depends for his virus, upon vaccine farms run for the profit of their owners, and he is compelled to rely upon these propagators and their assistants, residing in distant localities, for the reliability, the honesty and the purity of their products, whereas, in modified inoculation, he can provide his own material, and can calculate from his own information, to a minute, with regard to its freshness, and also in the matter of its purity.

7. Modified inoculation can be made stronger or weaker, to meet any required case or emergency; stronger, for example, in cases prudently needing a second or third protection, if an emergency should suggest repetitions.

8. One or two modified inoculations would answer for a lifetime, whereas, one-third of the re-vaccinated will make a response, if vaccinated with reliable virus every third year.

9. A vaccinated patient will actively respond to modified inoculation in a year, and even a smallpox patient, after recovery, will slightly, or positively, respond to modified inoculation, in the second, and even in the first, year.

10. To practice modified inoculation, it is simply necessary to obtain the smallpox lymph in the vesicular stage only, and admix the same thoroughly with from three to six drops of fresh, warm cow's milk, and proceed to operate precisely as for vaccination. Modified inoculation, thus practiced, is not communicated by contact or contagion.

The author contributed his army experience on this subject, in the first issue after the war, of the *New Orleans Medical and Surgical Journal,* owned and edited by the present Dean of the Medical Department of Tulane University, Professor S. E. Chaillé, M. D. And the writer, from that date to the present, in his private practice, when confronted with smallpox, has unvarially, successfully and satisfactorily practiced in every case, modified inoculation, feeling better pleased with the result from every additional experience had with the valuable remedy. And being the outgrowth of an experience reached in a grave and pressing emergency, where necessity was made the mother of this successful experiment, by a Confederate Surgeon, on Hatchie Island, surrounded by smallpox cases, the writer has deemed it proper and pertinent, before a Confederate reunion, to embody in his required report this important fact of his experience, in the interest of humanity, against the most loathsome of possible maladies.

The article referred to in the *New Orleans Medical and Surgical Journal* was forwarded to the Surgeon General of the United States Army a few years after its appearance, and the author holds his acknowledgment of same, and in *Gaillard's Medical Journal* of New York city, the author has contributed more than one article, setting forth his experience since the war. The subject has not been recently more vigorously pressed because the author did not desire a reputation which might associate him in the remotest manner with the care or treatment of smallpox cases, directly or indirectly.

Very respectfully and fraternally submitted,

C. H. TEBAULT, M. D.,

Brigadier General and Surgeon General U. C. V.'s, Staff of General J. B. Gordon.

—*Gaillard's Medical Journal,* January, 1899.

In connection with the above, Dr. Tebault sends us the following:

DEAR DR. DANIEL: In *Gaillard's Medical Journal,* August, 1883, on pages 146 and 147, the following will be found, which was sent to me by Dr. W. C. Powell, of Natchitoches, Louisiana, and which I forwarded to the above medical journal in question:

"On June 4th, of this year (1883), I was called to see Etienne A.'s daughter (black), aged 12 years. On entering the room of the patient I observed an extensive eruption on her face; on further examination I found both the anterior and posterior surfaces of nearly the entire body in the same condition. After carefully examining my patient and closely questioning the mother, I gained such information as to lead me to suspect I had a case of variola, discrete in form, to treat.

"As to the cause of this one case (only) having made its appearance in this locality was due to the following reasons, I judge, i. e., the patient herself had not gone off of the place, but a number of visiting friends from a plantation some miles below on the river (which had been visited by smallpox some weeks previous) came to spend a short time with patient's family. In all probability some of these persons themselves had been subjects of the disease, and, owing to their poverty, could not well afford to destroy the clothing, etc., and carried the malady, as it were, by 'fomites' to this house. My treatment of this case was according to the general plans and rules laid down by eminent authors for mild forms of the trouble.

On noticing carefully the patient, I observed that the eruption was of three days' standing, and a few of the eruptions looked as though they contained a sufficient quantity of the lymph to justify me in trying Dr. C. H. Tebault's (of New Orleans, La.) plan, that of modified inoculation (it seemed somewhat early, but I tried it nevertheless), which I proceeded to do at once, on six members of the family and two in an adjoining house. I did not see my patient again until the 8th, then in company with Drs. Charles Hamlin and Z. T. Gallion, who agreed in diagnosis with me: 'An undoubted case of variola, mild in form.' On examining the inoculated parties, found one who had the appearance of taking, but being in doubt we remodified inoculated this one as well as the others. Five days after I called, and found that all of the cases had taken well; one child had a few pustules on the forearm, also a few on the feet.

"The 22nd I visited my cases, and found the characteristic cicatrix as it should be, no untoward symptoms resulted from the modified inoculation, and no spread of the dreaded malady up to this date. I am pleased to state, that after I had paid my first visit to my patient, I spoke to my worthy friend and confrère, Dr. Charles Hamlin, and told him I feared I had a case of variola to treat very near us, and that to be on the safe side (knowing the liability of the household to take the disease) I had tried Dr. Tebault's mode, that of 'modified inoculation,' on several cases. He remarked, 'Well, you did exactly what was right; there is nothing like trying it, for those in the house with the case are more than apt to take smallpox, so give it a trial, and let's see what it will do.' So I did, and the result was a success. Should it be my lot again to be called to treat smallpox, I will use every endeavor to institute Dr. Tebault's plan of inoculation."

